# How Do Primary Care Organizations Rate Their Level of Organizational Health Literacy? Results of a Swiss Pilot-Study

**DOI:** 10.3390/ijerph192316139

**Published:** 2022-12-02

**Authors:** Anna-Sophia Beese, Dunja Nicca, Rebecca Jaks, Natascha Stuermer, Saskia Maria De Gani

**Affiliations:** 1 Center for Health Literacy, Careum Foundation, 8032 Zurich, Switzerland; 2 Epidemiology, Biostatistics and Prevention Institute, University of Zurich, 8001 Zurich, Switzerland; 3 Careum School of Health, Kalaidos University of Applied Sciences, 8006 Zurich, Switzerland

**Keywords:** health literacy, health literate organization, self-assessment tool, primary care, organizational health literacy, health professionals

## Abstract

Primary care organizations offer a suitable setting to promote organizational health literacy (OHL) since they are a crucial interface for patients and clients. However, a clear picture on the OHL situation in primary care is lacking. The aim of this study was to assess OHL in Swiss primary care organizations by evaluating (i) how they rate their level of OHL and which improvement measures they accordingly plan (organizational level), (ii) what knowledge and attitudes among health professionals in primary care regarding OHL exist (individual level) and (iii) how teams working in general practitioners’ practices and a home care service organization differ regarding OHL (organizational and individual level). The research design included an online survey (*N* = 74, health professionals) on the individual level and a checklist and intervention documentation (*N* = 10, primary care teams) on the organizational level. The study reveals a crucial demand regarding organizationally embedded OHL practices in the participating primary care teams, despite a rather comprehensive understanding of the concept. The greatest need for action was identified in strengthening health literacy of staff members, which underlines the necessity to develop interventions to systematically strengthen the health literacy of health professionals.

## 1. Introduction

Health literacy is the degree to which people have the knowledge, skills and the attitude to find, understand, assess and apply health-related information [1]. As an important socio-economic determinant of health, health literacy always needs to be considered in relation to social contexts and situated complexities, and is positively correlated with health behavior and health status [2,3,4]. Health literacy-oriented studies attest a low health literacy level for approx. 88% of US adults [5] and for 47% of the respondents of the European Health Literacy Survey (HLS-EU) [6,7]. Further, the recently conducted Health Literacy Survey Switzerland (HLS_19-21_-CH) showed that the difficulties in dealing with health-related information have increased somewhat over the last five years [8]. These numbers clearly highlight the need for improvement measures.

**Organizational health literacy (OHL)** supports patients, clients or relatives via social and healthcare organizations in accessing, understanding, evaluating and applying health information to make health-related decisions [9,10,11,12,13,14]. Organizational strategies to strengthen patients’ health literacy are, for example, to avoid medical jargon, break down information to patient-oriented small chunks or provide visualizations or pictograms to ensure a better understanding for patients [10,11]. In doing so, health literate organizations provide an important, yet underestimated, contribution to population health and thereby “[…] address demands and complexities of healthcare system[s]” [13] (p. 5).

Despite healthcare organizations’ promising role to improve patients’ health literacy, OHL measurement and improvement interventions are still scarce [9,13]. Therefore, with the aim of improving OHL in primary care, our team developed the so-called “Organizational Health Literacy Self-Assessment Tool for Primary Care” (OHL Self-AsseT) from 2019–2020 [2]. The tool can be used both as an assessment of OHL and as an intervention to improve OHL in primary care organizations. We used the developed tool [2] in a pilot-study with a small scalable unit of general practitioners’ practices (GPPs) and a home care service organization (HCSO) in Switzerland. Regarding the further development of the tool, the present study aimed to assess the level of OHL in primary care settings on two levels: (1) on the **individual level**, referring to the participating health professionals working in the field of primary care and (2) on the **organizational level,** referring to teams from one network of GPPs and a larger HCSO. Hence, the study evaluated (i) how primary care organizations rate their level of OHL and which improvement measures they accordingly plan (organizational level), (ii) what knowledge and attitudes among health professionals in primary care regarding OHL (individual level) exist and (iii) how the two settings in the field of primary care (GPP and HCSO) differ regarding OHL (organizational and individual level).

## 2. Methods

The present study is part of a larger mixed method study program to develop, evaluate and scale the OHL Self-AsseT for primary care in Switzerland [2,15]. The present study includes the elaboration of the knowledge and attitudes of health professionals towards OHL prior to the application of the OHL Self-AsseT. It analyzes health professionals’ OHL ratings of their organizational entities, describes for which of the six OHL dimensions health professionals identified the strongest need to act and which measures they planned to improve their level of OHL. The qualitative counterpart of this evaluation explores how the OHL Self-AsseT was implemented, understood and applied by primary care organizations and is published separately (the manuscript focusing on the qualitative evaluation analysis of this study program was submitted parallel to this *IJERPH* Special Issue “Health Literacy and Social Contexts”) [15].

### 2.1. The OHL Self-AsseT

The OHL Self-AsseT is a tool to simultaneously assess and improve OHL in primary care organizations. The first step of its development process is presented by our research team of De Gani and colleagues in a paper published in 2020 [2]. Shortly, the tool consists of three modules: the **manual (module 1)** provides a brief instruction on the concept of health literacy and OHL and explains how the self-assessment is performed and applied. The **checklist (module 2)** enables a collective self-assessment along six key dimensions of a health literate organization. The six dimensions include (i) “provide easy access to primary care services and facilitate navigation within”, (ii) “communicating in plain and easy to understand language”, (iii) “promoting health literacy of users”, (iv) “promoting health literacy of staff members”, (v) “incorporating health literacy into the management and organizational structure” and (vi) “promoting health literacy at care interfaces, networks and further activities of the organization” [2]. Each dimension consists of several sub-dimensions, which are further specified by multiple criteria. The degree of fulfillment of each criterion can range from “yes”, “rather yes”, “rather no”, “no” and “not applicable”. For each criterion, points can be assigned to automatically calculate the level of OHL for each of the six dimensions. The **handbook (module 3)** assists the participating organizations in defining and planning action and offers concrete ideas for measures and strategies to improve OHL. The handbook is also structured along the six dimensions of a health literate organization. The three modules build on each other and aim to support primary care providers in assessing the health literacy status of their organization. Furthermore, the modules support organizations in deriving potential need for action, defining common goals and planning, developing as well as implementing concrete measures regarding the promotion of OHL. Moreover, the tool aims at contextualizing OHL into existing organizational processes and structures.

### 2.2. Setting and Sample

A total of **four GPP teams** and **six organizational teams from one HCSO** in the canton of Zurich participated in this pilot-study. These ten organizational teams were a convenience sample, specially recruited for this pilot-study through contacting network partners of the project owners Careum Foundation Zurich and the Health Department of the Canton Zurich (represented by the Epidemiology, Biostatistics and Prevention Institute of the University of Zurich). These network partners included a larger network of GPPs, consisting of 62 practices and an HCSO, composed of 56 nursing teams. Both settings—the GPP and the HCSO—are vital primary care settings in Switzerland with the core characteristics of patient interactions. The research team asked a managerial person in both the GPP and the HCSO network to recruit teams within their organization to apply and implement the tool. The inclusion criteria for participation in the pilot-study were that the organizations (a) were interested in promoting OHL at multiple organizational levels, and that (b) at least two to five health professionals were willing to use the tool. In the beginning, eight teams from the HCSO and four GPPs agreed to participate. However, before the implementation started, two HCSO teams withdrew their participation due to the increasing workload amid the SARS-CoV-2 pandemic. Essentially, six HCSO teams and four GPPs participated in this study.

The GPPs’ staff included medical doctors, medical practice assistants (MPAs, i.e., clinical administrators with training to perform common medical procedures) and MPAs in training. From the 67 health professionals employed at the four GPPs, 36 health professionals participated in the study. The employees of the six participating HCSO teams included registered nurses, nursing assistants as well as advanced practice nurses (APNs, i.e., nurses with a master’s degree). The focus of the participating HCSO lies in nursing, care services and housekeeping services in the patients’ and clients’ homes. From the 54 health professionals employed in the six HCSO teams, 38 health professionals participated in the study. Thus, in total 74 individual health professionals from ten organizational teams participated in the study. The governance of the participating HCSO teams is characterized by heterarchical structures and rather autonomous, self-managed teams, inspired by the Dutch Buurtzorg model [16]. However, no specific focus was put on the governance mode during the recruitment process of the participating teams, rather the trustful partnership with the HCSO and their motivation to strengthen OHL in the primary care setting were key indicators to recruit the HCSO teams.

### 2.3. Data Collection

To assess knowledge and attitudes towards OHL on the individual level, an **online survey** (16 items) was conducted prior to the intervention of the OHL Self-AsseT from January 2020 to June 2020. The authors sent the survey link to a lead person of the GPPs and the HCSO. The lead persons sent the survey link to all employed health professionals (121 health professionals) of the participating GPP and HCSO teams. To ensure the study participants’ anonymity, the following mechanism was applied: first, the online survey was anonymously filled out by the participants and the project team was not able to match the filled-out surveys to specific persons. Second, no list with names of the employed health professionals in the participating primary care teams was provided, thus, the project team was not aware of the participants’ identities. Third, all the documents of the OHL Self-AsseT concerning the pilot-organizations were filed in folders that were only accessible to the research team and were neither used for any other reasons than for the purpose of this study, nor were they available for other officials or employees outside the research team. The online survey consisted of a self-developed questionnaire in the German language and comprised 16 questions, which were clustered into five parts. The first part included seven questions about socio-demographic characteristics. The second part covered four questions about the term health literacy: whether participants had heard of the term and concept of OHL before, and whether they could properly classify it. The third part included one question about the knowledge and the fourth part one about the attitude of the participating health professionals towards OHL. Participating health professionals were able to rate their knowledge on a six-point Likert scale, ranging from (1) very comprehensive, (2) comprehensive, (3) rather comprehensive, (4) rather low, (5) low, (6) very low. For the analysis, we dichotomized the scale into “comprehensive” (response options 1–3) and “low” (response options 4–6). Regarding the attitude towards health literacy, participating health professionals could assess whether they agreed with a statement or not, also based on a six-point Likert scale ranging from (1) fully agree to (6) fully disagree. In a second step, we combined the responses into (1) “agree”, including option 1 to 3, and (2) “not agree”, including option 4 to 6. The fifth part comprised three questions about the completion of trainings in this field and the degree of involvement within the pilot-project of the OHL Self-AsseT.

To evaluate the level of OHL, we used **data from the checklist** (module 2, 75 items) of the OHL Self-AsseT. The checklist was not only used to assess the level of OHL but was at the same time also applied by all ten participating teams as an intervention to improve their level of OHL. The intervention lasted over a period of seven months (from May 2020 to November 2020). The application of the OHL Self-AsseT, i.e., the assessment and the intervention, took place in interdisciplinary and cross-hierarchical groups of health professionals, with the aim of incorporating different experiences and perspectives into the assessment and planning of the improvement measures. Therefore, in all participating primary care teams, so-called **self-assessment teams** were formed [15]. Wherever possible, the self-assessment team consisted of people from different professional groups (e.g., medical doctors, medical practice staff, nursing staff), hierarchical levels (e.g., organizational management, team management, staff) and employment relationships (e.g., full-time, part-time, self-employed, employed). Within the self-assessment team, a coordinating person was selected to provide leadership and coordinate the self-assessment process. This self-assessment process included (i) filling in the checklist individually (approx. 30 min), (ii) reaching a collective consensus for the organization within the self-assessment team (approx. 90 min) and filling in the checklist Excel spreadsheet and (iii) identifying need for action and planning at least two OHL development goals as well as respective measures to improve the OHL and fill in those insights in a specifically prepared Word document. To evaluate these planned and at least partially implemented improvement measures, we used the data from this **intervention documentation** of the self-assessment teams. All the completed materials were forwarded to the research team via email after the intervention. Table 1 provides an overview on the sampling and data collection.

### 2.4. Data Analysis

For data analysis, we used frequency and mean analyses on a univariate level, and cross-tabulations and significance tests on a bivariate level using IBM SPSS Statistics 27 (IBM Corp. Armonk, NY, USA). Continuous variables were described using means (M) and standard deviations (SD).

First, to gain a more thorough understanding of the level of OHL in primary care settings on an individual level, descriptive data analysis of knowledge and attitudes of health professionals in the primary care setting towards OHL prior to the application was performed. Frequencies of socio-demographic characteristics of the responding health professionals were analyzed through frequency counts and descriptive statistics. Additionally, a χ^2^ test for association was conducted between the two settings (GPP and HCSO) for knowledge and attitudes towards health literacy. Second, to evaluate how primary care organizations rated their level of OHL, a descriptive analysis for the overall sample of health professionals was conducted. In addition, the Mann–Whitney U test for independent samples was then applied to determine whether the tendencies in the ratings of the two independent samples of the GPPs and the HCSO differed. Furthermore, a descriptive analysis revealed those areas in which health professionals most often set goals and planned measures to strengthen OHL. The internal reliability of the statistical instruments was assessed using Cronbach’s alpha coefficient. The reported *p*-values are based on the two-tailed test, and the level of statistical significance for all tests was established at *p* < 0.05.

## 3. Results

### 3.1. OHL from an Individual Level Perspective

The following sub-sections elaborate on the knowledge and attitudes of health professionals in the participating primary care organizations towards (organizational) health literacy.

#### 3.1.1. Socio-Demographic Characteristics

The respondents’ socio-demographic characteristics are summarized in Table 2. The participating health professionals (*N* = 74) were mostly female (91.9%) and between 41 and 65 years old (47.3%). Compared to the participating health professionals of the HCSO, more than one third of the participating health professionals of the GPPs held a managing function. Furthermore, while the majority of health professionals of the GPPs worked more than 80%, most of the HCSO staff reported a full-time equivalent (FTE) between 61 and 80%.

#### 3.1.2. Knowledge of Relevant Health Literacy-Related Terms

**Figure 1:**
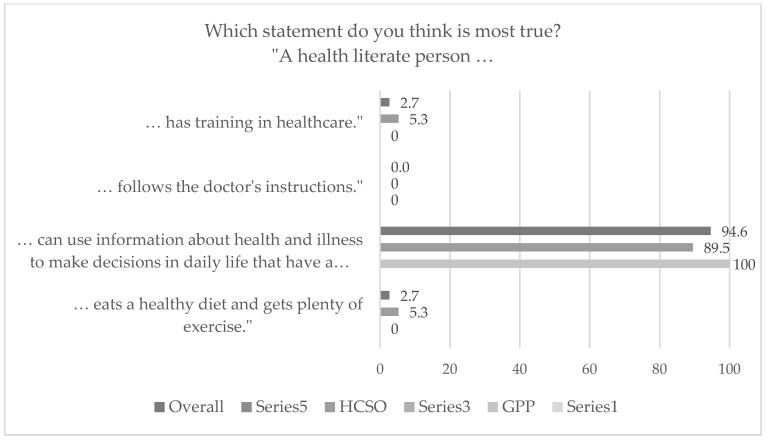
Primary care providers’ understanding of the term health literate person (*N* = 74 overall, data in %). HCSO = home care service organization; GPP = general practitioners’ practice.

Most of the respondents (77% overall, i.e., all ten organizational teams combined) reported that they had heard the term health literacy before. In the six organizational teams from the HCSO, the proportion of those who had heard of the term tended to be higher (78.9%) compared to the four GPPs (75%). However, the results showed no statistically significant difference between GPPs and HCSO (*p* = 0.135). Concerning the understanding of the term “health literate person”, almost all participants (94.6%) defined it correctly as “someone who can use information about health and illness to make decisions in daily life that have a positive impact on health” (see Figure 1). No statistically significant difference was found in this regard between the two primary care settings (*p* = 0.530).

#### 3.1.3. Knowledge of Health Literacy and Attitudes towards OHL

**Figure 2:**
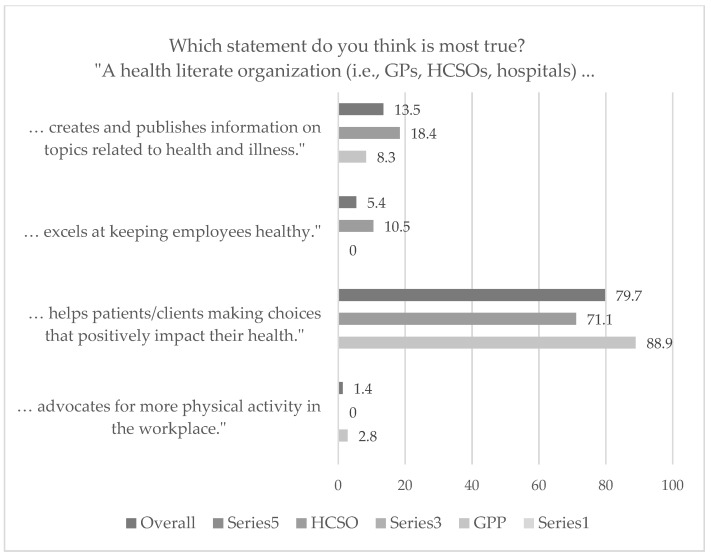
Primary care organizations’ understanding of the term health literate organization (*N* = 74 overall, data in %). HCSO = home care service organization; GPP = general practitioners’ practice.

Regarding organizational health literacy, most of the respondents correctly agreed on a **health literate organization** being an organization which “helps patients/clients making decisions that positively impact health”. A closer look at the two analyzed primary care settings reveals that the outcomes between the organizational teams of the HCSO and GPPs tend to vary slightly. While 28.9% of the health professionals from the HCSO thought that a health literate organization designs and distributes information on health-related topics or excels at improving staff’s health, only 8.3% of the GPPs’ health professionals did so (see Figure 2). However, these differences were not statistically significant (*p* = 0.073).

The question of “how health professionals in both settings assessed the **current health literacy status of the general Swiss population**” could be marked by seven statements regarding the Swiss population either as true or false. The correct answers based on evidence from a Swiss study [17] are marked with a * for each statement in Table 3. Results show a significant difference between the two settings and the assessment of health literacy of the Swiss population for statements 1, 3 and 4. More than half (52.6%) of the health professionals from the HCSO agreed that dealing with health information is difficult for most of the population, while three quarters (75%) of the health professionals from the GPPs marked the statement as true (statement 1, *p* = 0.046, see Table 3). Most of the participants from both settings (75.7%) correctly acknowledged that more than half of the population finds it difficult to assess the advantages and disadvantages of treatments (statement 2, *p* = 0.895), while considering it false (77%) to be easy for individuals to assess how trustworthy health information in the media is (statement 3, *p* = 0.001). The highest approval overall (81.1%) for a statement applying to the Swiss population was about the challenges for older people to deal with information on health and illness (statement 4, *p* = 0.002). Regarding statement 5 (*p* = 0.153), the participating GPPs agreed slightly more strongly that individuals with higher health literacy rate their health status more positively. Furthermore, most of the participating health professionals (70.3%) correctly stated that individuals with higher health literacy levels are less likely to go to the doctor (statement 6, *p* = 0.112) and that individuals with higher levels of education find it easier to understand health-related information (statement 7, *p* = 0.242).

**Figure 3:**
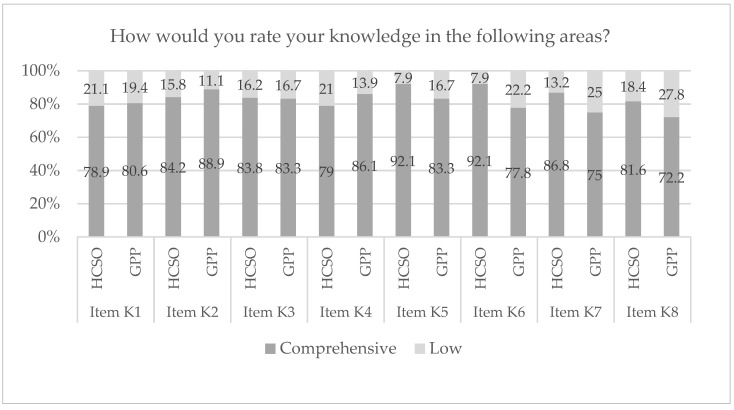
Health professionals’ knowledge regarding health literacy in primary care. Legend: Item K1 = tools to help patients/clients navigate medical offices or hospitals; Item K2 = interview techniques to help patients/clients and their families understand interview information; Item K3 = ways to help patients/clients with language difficulties to articulate themselves; Item K4 = sources of trustworthy and easy-to-understand patient information; Item K5 = approaches to help patients/clients make decisions about their care; Item K6 = ways to help patients/clients make lifestyle changes; Item K7 = services to help patients/clients manage their conditions; Item K8 = measures to help patients/clients transition to other care providers; HCSO = home care service organization; GPP = general practitioners’ practice.

A further question concerned the **knowledge of health professionals on health literacy** in general. Health professionals rated their knowledge as rather comprehensive, with values varying between 72.2% and 92.1% (see Figure 3). There were no statistically significant differences between the two organizational settings of the HCSO and GPPs in terms of “tools to help patients/clients navigate medical offices or hospitals” (item K1, *p* = 0.812), and “ways to help patients/clients with language difficulties to articulate themselves” (item K3 *p* = 0.610). In addition, the two settings seem to have similar ratings of knowledge on “interview techniques to help patients/clients and their families understand interview information” (item K2, *p* = 0.615) and on “sources of trustworthy and easy-to-understand patient information” (item K4, *p* = 0.392).

In contrast, it could be observed that the health professionals of the HCSO rated their knowledge as more comprehensive than those from the GPPs regarding the remaining four items. Health professionals of the HCSO were more likely to “know approaches to help patients or clients make decisions about their care” (item K5, *p* = 0.016, 92.1% vs. 83.3%) and know “ways to help patients/clients make lifestyle changes” (item K6, *p* = 0.045, 92.1% vs. 77.8%) than those of GPPs. Similarly, “knowledge of services to help patients or clients manage their conditions” (item K7, *p* = 0.001 86.8% vs. 75%) shows a significant difference between the two settings. No statistically significant differences between health professionals of the participating GPPs and the HCSO were found for knowledge on “measures to help patients/clients transition to other care providers” (item K8, *p* = 0.154).

**Figure 4:**
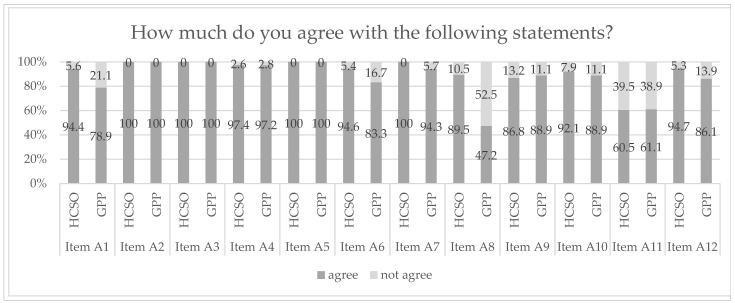
Health professionals’ attitudes towards health literacy in primary care. Legend: Item A1 = Health literacy is relevant for organizations such as GPs, HCSOs and hospitals and not a trendy topic; Item A2 = When talking to patients and their relatives, it is important to use simple and easy-to-understand language; Item A3 = When talking to customers it is important to make sure that they understand all the information; Item A4 = In everyday practice, it is important to respond to different language needs of patients; Item A5 = Advantages and disadvantages of different treatment options should be explained to patients in a comprehensive and easy-to-understand manner; Item A6 = Informing patients/clients about what they can do themselves for their own health is the task of GPs/HCSOs; Item A7 = It is important to support patients/clients with concrete offers and information in dealing with their illness; Item A8 = The opinion of patients and clients should always be taken into account when creating new offers, materials and documents in the GP/HCSO; Item A9 = It is part of the role of GP/HCSO staff to help patients/clients navigate the “jungle of the healthcare system”; Item A10 = It is important to assist patients/clients transition to other care providers; Item A11 = It is important to contact patients between visits to ask if they have questions or need assistance; Item A12 = Healthcare providers should work together to provide health literacy services for patients/clients (e.g., training or information materials); HCSO = home care service organization; GPP = general practitioners’ practice.

The results of the attitudes of health professionals in primary care settings towards OHL are illustrated in Figure 4 (items A1 to A12) (item A1 was originally asked in the form of a negative question, which in the best case would not be agreed to. To analyze the data in a more congruent way, we adapted the question during the coding process). Most health professionals agreed that “health literacy is relevant for organizations such as GPs, HCSOs and hospitals and not a trendy topic” (item A1, *p* = 0.451). The importance of using simple and easy-to-understand language (item A2, *p* = 0.076), of ensuring whether patients and clients have understood the information (item A3, *p* = 0.076) and explaining advantages and disadvantages of different treatment options in a comprehensive manner (item A5, *p* = 0.867) was unanimously agreed on by health professionals of both settings. Similar, yet with slightly less overall agreement, the health professionals from both settings approved that in everyday practice, it is important to respond to different language needs of patients (item A4, *p* = 0.749) and that it is part of the role of GPP/HCSO staff to help patients/clients to navigate the “jungle of the healthcare system” (item A9, *p* = 0.222). Furthermore, statistically significant differences between the settings could be observed for all the other statements with which health professionals of the HCSO agreed more often than those of the GPPs. While most of the statements were agreed to by both the GPPs and the HCSO, item A8 and item A11 showed a different response pattern. Nearly 90% of the health professionals in the HCSO agreed that “the opinion of patients and clients should always be considered when creating new offers, materials and documents”, yet less than half of the health professionals in GPPs agreed with this statement (item A8, *p* < 0.001). The importance of contacting patients between visits to ask if they have questions or need assistance (item A11, *p* = 0.505) also received less approval, but this time from both settings equally (60.5% vs. 61.1%).

### 3.2. OHL from an Organizational Level Perspective

The following sub-sections describe how primary care organizations rated their level of OHL and what measures they subsequently planned to improve OHL in those dimensions where they identified need for action.

#### 3.2.1. Primary Care Organizations Rating Their Level of OHL

The following results originate from the **checklist** (module 2 of the OHL Self-AsseT), completed by each self-assessment team. The results illustrate how they **assessed their level of OHL** along the six dimensions of a health literate organization (see Table 4). 

The results show that the participating GPPs tended to have a lower need for action in four out of the six dimensions (dimensions 1, 2, 4 and 6) compared to the participating HCSO teams. Thus, the GPPs seem to have a slightly higher level of self-reported OHL. The self-assessment teams from both primary care settings identified the greatest need for action in “promoting health literacy of staff members” (dimension 4). While “providing easy access to primary care service and facilitate navigation within” (dimension 1) was identified as the OHL dimension with the least need for action by GPPs, the HCSO teams assessed the lowest demand for further interventions in “promoting health literacy of their users” (dimension 3).

#### 3.2.2. Planned Measures to Promote and Strengthen Health Literacy

Overall, the ten self-assessment teams planned 33 improvement measures to foster and promote their level of OHL (see Table 5). The self-assessment teams themselves decided in which of the six OHL dimensions the planned measures best fit and categorized the measures accordingly. Most improvement measures were planned to enhance the health literacy of staff members (dimension 4), which corresponds to the dimension with the greatest need for action, followed by measures to “provide easy access to primary care service and facilitate navigation within” (dimension 1). In dimension 4, the health professionals planned health literacy-related improvements of two types: first, they planned measures that focused on developing health literacy-oriented guidelines. The guidelines were planned to be developed by medical doctors either for patients or clients, or for internal usage targeting peer health professionals and focusing on communication aspects. Measures to develop guidelines were primarily planned by the participating GPPs. Second, the planned measures targeted practices of interacting and communicating in team meetings. Such practices included (i) an active and regular discussion of health literacy-related topics in meetings, prepared and presented by team members, (ii) short health literacy refreshers during meetings and (iii) the improvement of team culture. In contrast to the health literacy-oriented guidelines, measures to strengthen health literacy via team interaction were predominantly planned by teams of the HCSO.

## 4. Discussion

This study of the OHL Self-AsseT reveals findings in terms of how primary care organizations in Switzerland assess and rate their level of OHL and their corresponding knowledge and attitudes. The results of this pilot-study on primary care professionals and organizational teams indicate that there is a basic understanding of the term health literacy. At the same time, concrete practices and viable organizational structures in primary care to promote OHL still have great potential for improvement. Furthermore, need for action according to the teams’ self-reported OHL was particularly identified regarding the promotion of health literacy of staff members.

### 4.1. Health Literacy—An (un)Familiar Concept in the Swiss Primary Care Setting?

The recently conducted HLS_19-21_-CH demonstrated that most Swiss inhabitants face multiple difficulties when it comes to adequately dealing with health information in order to make well-informed health-related decisions [8]. These difficulties also include finding out one’s own rights as a patient, to understand healthcare reforms, or to navigate healthcare systems [8]. To tackle these difficulties, primary care organizations and their professionals play a crucial role in strengthening health literacy, i.e., supporting patients and clients in and from different social contexts to adequately deal with health information [10,18,19]. An essential requirement to fulfill this task is that health professionals have the therefore necessary knowledge, attitudes, skills and conditions.

The present study indicates that health literacy both as a term and concept is rather well known among the participating Swiss primary care health professionals. Our data reveal that while most of the health professionals have heard of the term health literacy, there is still a minor, yet considerable number in both primary care settings who had never heard of the term before (25% of the employees of the HCSO and 18.4% of the GPPs). Accordingly, the health professionals rated their own knowledge in terms of health literacy mostly as “rather comprehensive” or “comprehensive”. These results offer two important insights on the level of health literacy in primary care: first, the rather comprehensive ratings of health professionals regarding their knowledge of health literacy indicate the promising potential of health professionals in primary care to actively support patients in dealing with health information. Second, health professionals in primary care may, at least to some extent, be sensitized for the multi-dimensional concept of health literacy, despite the lack of health literacy as a subject both in the curricula of Swiss health professionals as well as in the curricula of the overall education system [20]. Notwithstanding scattered indicators of a rather comprehensive individual understanding of the concept, the study emphasizes a strong need for action from an organizational perspective. The self-assessment of the level of OHL indicates that according to the participating teams of both settings, the promotion of health literacy of the workforce, at interfaces and networks of healthcare as well as organizational practices, requires further improvement.

### 4.2. Health Literacy as an Inherent Task of Primary Care Providers

The applied organizational lens of this study demonstrates that respondents perceived health literacy as the process of informing and supporting patients or clients on what they can do themselves for their own health as an inherent task of primary care. Furthermore, most of the health professionals saw it as their role as an employee of a GPP or HCSO to help patients or clients situated in their individual social context to navigate through multiple complexities of the healthcare system and to support patients with concrete services or information in dealing with their illness. Furthermore, the present findings illustrate that the potential of OHL is already partly anchored in the participating organizational teams since essential tools and elements are used in parts of their daily work. This seems to be particularly true for the HCSO as indicated by the answers to the question of health literacy and its importance: while only a few health professionals from the HCSO believed health literacy to be a mere trend and of minor importance, around four times as many did so from the GPPs (Figure 4). Thus, the results of the participating teams of the HCSO indicate a good understanding of what health literacy is, why it is important and how it can be applied for the benefit of patients or clients. Nevertheless, overall, the GPPs tended to report slightly higher levels of OHL. A possible explanation for the different attitudes regarding health literacy, OHL and its importance could be that the participating HCSO has recently transformed its organizational structure towards the establishment of lean processes for more continuity and accessibility, better neighborhood networking and knowledge management—based on the “Buurtzorg model” from the Netherlands [16,21,22]. At the heart of this organizational development process are self-organized teams without standardized management hierarchies and characterized by their small size and great proximity to patients, clients and relatives. This new structure and the patient-oriented processes might have paved the way for an agile response to local conditions, local networking and better customer continuity and accessibility, as described in the literature [23]. With the transformation to this organizational form, the health professionals from the participating HCSO teams might have different prerequisites regarding their attitudes towards health literacy and OHL, as patient and client continuity and orientation are also an important component in the “Buurtzorg model” [16,22]. Furthermore, their heterarchical form based on team processes and shared decision-making practices may further have encouraged them to integrate and embed health literacy-related tools into their daily work routines rather independently, to enact communication practices among team members and in interaction with patients or clients. Following such an approach allows healthcare providers to strengthen both patient orientation and health literacy in a targeted manner in addition to their daily value creation activities. Despite the rather good organizational preconditions to implement OHL, particularly in the HCSO, the present findings also show some challenges and action required for both settings.

For instance, it remains rather ambiguous how patient orientation combined with the promotion of health literacy is applied in the context of primary care. Health professionals reported the importance of involving patients regarding the development of information material or communication processes. However, statements regarding the concrete involvement of patients in decision-making processes received very little overall agreement. These findings are in line with the results of the self-assessment, especially of dimension 3 “promoting health literacy of users”, where the teams of both settings stated a need for action. In addition, at least in the HCSO setting health professionals seem to be sensitized for the importance of the active inclusion of needs and opinions of patients and clients in the development of new services or information materials. The reason therefore might again be the organizational structure of the HCSO that promotes strong patient orientation and working “inside-out” with clients as the core of all work-related practices [22]. Patient orientation is therefore already strongly practiced in the HCSO context and shaped together with the patients or clients [24]. In sum, the results underline the importance of patient-oriented action to strengthen a health literacy-oriented organizational environment in the long run.

### 4.3. The OHL Self-AsseT as a Tool to Support Health Literacy (Of Staff Members)

Applying the OHL Self-AsseT enabled participating organizational entities to strengthen their OHL by self-assessing their level of OHL, identifying a need for action and defining goals and measures for improvement. At the same time, the tool also seemed to encourage the participating health professionals to reflect upon their own professional health literacy. The greatest need for action overall was identified in strengthening health literacy of staff members. At first sight, this finding seems rather surprising regarding the fact that the majority of the participating health professionals rated their knowledge and attitudes towards health literacy as comprehensive in the online survey prior to the OHL Self-AsseT intervention. However, a closer look at the data reveals some important distinctive features. The OHL dimension 4 “promoting health literacy of staff members” includes three sub-dimensions (see also Table 4). While the sub-dimension on the know-how and professional competences of health professionals was rated rather sufficient, a stronger need for action was identified regarding personnel development and the staff members’ health. These findings indicate that health professionals in primary care seem to perceive health literacy as a broader concept beyond knowledge and attitude on certain aspects of this concept. These findings are in line with recent research on health literacy regarding professional health literacy [10]. The health literacy of health professionals should be improved not only in respect of a more adequate and patient-oriented communication but also regarding a more frequent information sharing among health professionals or between health professionals and patients. In fact, measures on professional health literacy might also strengthen professionals’ own health. Since studies indicate that a persons’ health is partly determined by their personal health literacy, the OHL Self-AsseT could contribute to enhancing both health literacy and healthy staff in primary care [25,26]. Healthier staff members in turn tend to be more satisfied with their job and are more likely to stay in the job [27,28]. Hence, the promotion of health literacy of staff members also bears some potential to curb the current staff shortage. At the same time, these findings are not limited to the primary care setting but provide useful guidance beyond the boundaries of primary care towards other social and healthcare settings. In sum, the results clearly indicate that further research not only must be conducted to explore the professional health literacy of health professionals but also to investigate how organizational practices and processes can be designed to support the promotion of health literacy of staff members and the entire organization—in primary care and beyond.

## 5. Limitations and Strengths

The time frame for the OHL Self-AsseT pilot-study was during the COVID-19 pandemic, when health professionals in primary care worked at the frontline to stem the pandemic. Thus, the recruiting process led to a small sample size, which limited the study analysis to be predominantly descriptive and limits generalizable statements regarding the Swiss primary care setting in general. Nevertheless, since this quantitative study is based on the pilot-project to develop, test and evaluate a new self-assessment tool and—regarding the field of implementation science—seen as the smallest scalable unit [29], we strongly believe our findings to provide valuable insights on how the participating teams rate their OHL, despite the small sample size. Furthermore, the teams participating in the study might have been more aware of the term and concept of health literacy than those who did not. Therefore, the participating GPPs and the HCSO were probably rather well sensitized about OHL, and the conclusions on knowledge and attitudes as well as levels of OHL in these primary care organizations might be overestimated. Another limitation is the self-reported data, which always carry the risk of reporting bias and social desirability. However, we see no reason why health professionals would misreport on their knowledge on and attitudes towards OHL as well as concerning their organizations. This is because the online survey was conducted anonymously and all results from the online survey as well as from the checklist were reported anonymized as well and were not traceable to the individuals. Furthermore, no incentive to benchmark with other organizations existed. Regardless of the rather positive OHL ratings of the participating teams, the present study still reveals an urgent need for action for primary care organizations to strengthen their OHL, especially in certain dimensions. This need is underlined by further qualitative findings. As the study at hand was embedded in a larger study program, additional auspicious findings from this study can be found elsewhere [15]. Both quantitative and qualitative evaluation studies combined provide a new orientation on how health professionals rate their OHL in primary care and on implementation processes to further develop and scale up the OHL Self-AsseT as a practice-oriented tool to strengthen OHL in healthcare.

## 6. Conclusions

The study at hand adds new insights to the increasing research on OHL in the setting of primary care in Switzerland on an individual and an organizational level. It focused on how health professionals in primary care rate their knowledge and attitudes towards OHL prior to the use of a self-assessment tool, i.e., the OHL Self-AsseT. The findings show that the term and concept of health literacy were already known by the health professionals of the participating primary care organizations to some extent. Regarding the self-assessment of OHL, the participating primary care organizations in both settings (GPP and HCSO) identified various needs for action. They both identified the greatest need in the dimension of health literacy promotion of the workforce. Consequently, further studies should focus on health professionals’ individual and professional health literacy. While the participating GPPs perceived the incorporation of health literacy into the management and organizational structure of the GPPs as the second most important dimension, the teams from the HCSO did so for providing easy access and navigational support. Our results show that the developed OHL Self-AsseT could be a useful tool to (1) assess OHL in primary care organizations, and (2) to implement first initiatives and measures to strengthen OHL. The present study—as part of a larger study program [2,15]—demonstrates that the sustainable implementation of health literacy into patient pathways as well as into organizational structures and processes contains great potential to enhance healthcare quality and coordination in the long term. Nonetheless, further research in this area is necessary.

## Figures and Tables

**Table 1:** Sampling and data collection.

Level	Timepoint	Method	Sample
Individual level	Prior to the intervention	Online survey	74 health professionals
Organizational level	During the intervention	Checklist (i.e., module 2 of the OHL Self-AsseT ^1^) Intervention documentation	10 teams (six teams from the HCSO and four GPPs)

^1^ OHL Self-AsseT = Organizational Health Literacy Self-Assessment Tool for Primary Care; HCSO = home care service or-ganization; GPP = general practitioners’ practice.

**Table 2:** Socio-demographic characteristics of the sample of the online survey (*N* = 74).

Sample Characteristics		HCSO *N* = 38 (%)	GPP *N* = 36 (%)	Overall *N* = 74 (%)
Gender, *n* (%)	Male	1 (2.6)	5 (14.0)	6 (8.1)
	Female	37 (97.4)	31 (86.0)	68 (91.9)
Age, *n* (%)	Younger than 20 years	1 (2.6)	4 (11.0)	5 (6.8)
	Between 20 and 40 years	11 (28.9)	22 (61.0)	33 (44.6)
	Between 41 and 65 years	26 (68.4)	9 (25.0)	35 (47.3)
	Older than 65 years	0 (0.0)	1 (3.0)	1 (1.4)
Function GPP ^1^, *n* (%)	Medical doctor	-	9 (25.0)	-
	Practice manager	-	1 (2.8)	-
	Medical practice assistant (MPA)	-	23 (63.9)	-
	Apprentice	-	2 (5.6)	-
	Not specified	-	4 (11.1)	-
Function HCSO ^1^, *n* (%)	Nursing specialist	11 (28.9)	-	-
	Healthcare specialist	9 (23.7)	-	-
	Home nurse	4 (10.5)	-	-
	Nursing assistant Swiss Red Cross	8 (21.1)	-	-
	Housekeeping assistant	9 (23.7)	-	-
	Apprentice	1 (2.6)	-	-
	Not specified	4 (10.5)	-	-
Managing function, *n* (%)	Yes	2 (5.3)	13 (36.0)	15 (20.3)
	No	35 (92.1)	23 (64.0)	58 (78.4)
	Not specified	1 (2.6)		1 (1.4)
FTE, *n* (%)	From 0% to 40%	1 (2.6)	1 (2.8)	2 (2.7)
	Between 41% and 60%	6 (15.8)	8 (22.2)	14 (18.9)
	Between 61% and 80%	20 (52.6)	6 (16.7)	26 (35.1)
	More than 80%	11 (28.9)	20 (55.6)	31 (41.9)
	Not specified	0 (0.0)	1 (2.8)	1 (1.4)
Years in the organization, *n* (%)	Less than 2 years	11 (28.9)	7 (19.4)	18 (24.3)
	Between 2 and 5 years	7 (18.4)	16 (44.4)	23 (31.1)
	Between 6 and 10 years	5 (13.2)	8 (22.2)	13 (17.6)
	More than 10 years	15 (39.5)	5 (13.9)	20 (27.0)
Work experience, *n* (%)	Still in training	1 (2.6)	2 (5.6)	3 (4.1)
	Less than 5 years	5 (13.2)	5 (13.9)	10 (13.5)
	Between 5 and 14 years	13 (34.2)	19 (52.8)	32 (43.2)
	Between 15 and 25 years	8 (21.1)	7 (19.4)	15 (20.3)
	More than 25 years	8 (21.1)	3 (8.3)	11 (14.9)
	Not specified	3 (7.9)	0 (0.0)	3 (4.1)

^1^ Multiple choice possible; FTE = full-time equivalent; HCSO = home care service organization; GPP = general practitioners’ practice.

**Table 3:** Health professionals’ assessment of statements regarding health literacy of the Swiss population.

No.	Which Statements Do You Think Apply to the Swiss Population?	HCSO (*N* = 38)		GPP (*N* = 36)		Overall (*N* = 74)	
		True	False	True	False	True	False
1	Dealing with health information is difficult for most of the population *. (true)	52.6%	47.4%	75%	25%	63.5%	36.5%
2	More than half of the population finds it difficult to assess the advantages and disadvantages of treatments. (true)	76.3%	23.7%	75%	25%	75.7%	24.3%
3	For many individuals, it is easy to assess how trustworthy health information in the media is *. (false)	39.5%	60.5%	5.6%	94.4%	23%	77%
4	It is particularly difficult for older people to deal with information on health and illness *. (true)	94.7%	5.3%	66.7%	33.3%	81.1%	18.9%
5	Individuals with higher health literacy rate their health status more positively. (true)	68.4%	31.6%	80.6%	19.4%	74.3%	25.7%
6	Individuals with higher health literacy are less likely to go to the doctor. (true)	63.2%	36.8%	77.8%	22.2%	70.3%	29.7%
7	Individuals with higher levels of education find it easier to understand information about health and illness. (true)	76.3%	23.7%	63.9%	36.1%	70.3%	29.7%

* Statements show a significant difference between the two settings. HCSO = home care service organization; GPP = general practitioners’ practice.

**Table 4:** Analysis of the OHL Self-AsseT checklist filled out by the self-assessment teams of the home care service organization (HCSO) and the general practitioners’ practices (GPPs). The values range between 0%, indicating no fulfillment of the corresponding criteria, and 100%, indicating that the criteria were completely fulfilled by the organization, according to the self-assessment teams.

Dimensions of a Health Literate Organization	HCSO (*N* = 6) in %		GPP (*N* = 4) in %		Overall (*N* = 10) in %	
	M ^1^	SD ^1^	M	SD	M	SD
**1. Provide easy access to primary care service and facilitate navigation within**	**65.9**	6	**84.5**	6	**73.3**	11
1.1 Contact (5 items)	74.5	12	88.3	7	80.0	12
1.2 Navigation within the primary care service (2 items)	44.5	12	75.0	8	56.7	19
**2. Communicating in plain and easy-to-understand language**	**74.3**	13	**75.0**	9	**74.6**	12
2.1 Oral communication (9 items)	74.7	16	75.9	4	75.2	13
2.2 Written communication (7 items)	73.8	14	73.8	15	73.8	15
**3. Promoting health literacy of our users**	**76.4**	18	**73.6**	14	**75.3**	16
3.1 Empowering our users to use health information (4 items)	82.0	23	75.0	17	79.2	21
3.2 Promoting an active role and self-management of our users (8 items)	73.6	17	72.9	16	73.3	17
**4. Promoting health literacy of staff members**	**58.9**	11	**60.6**	18	**59.6**	14
4.1 Know-how and professional competence (3 items)	79.6	23	58.3	32	71.1	29
4.2 Personnel development (10 items)	52.2	19	57.5	18	54.3	19
4.3 Staff members’ health (2 items)	61.1	18	79.2	14	68.3	19
**5. Incorporating health literacy into the management and organizational structure**	**73.5**	14	**65.6**	9	**70.3**	13
5.1 Health literacy as an organizational responsibility (4 items)	80.6	15	52.1	15	69.2	20
5.2 Health literacy as a development goal (4 items)	66.7	10	70.8	7	68.3	9
5.3 Organizational culture (4 items)	75.9	17	68.8	14	73.1	15
5.4 User involvement—feedback (4 items)	71.3	22	70.8	14	71.1	19
**6. Promoting health literacy at care interfaces, networks and further activities of the organization**	**66.2**	11	**73.2**	11	**69.0**	12
6.1 Care interfaces (3 items)	93.5	7	69.5	12	83.9	15
6.2 Networking and further activities (6 items)	50.0	14	75.7	11	60.3	18
**All dimensions** (75 items)	**69.2**	6	**72.1**	8	**70.3**	5

^1^ M = mean; SD = standard deviation, weighted by number of items.

**Table 5:** Overview on the planned improvement measures per dimension in teams of the GPPs and the HCSO.

OHL Dimension	Planned Measures by Self-Assessment Teams of the GPPs	Planned Measures by Self-Assessment Teams of the HCSO	Overall No. of Planned Improvement Measures
1. Provide easy access to primary care service and facilitate navigation within	“Labeling access for the underground parking lot” (GPP-1)	“At the inter-disciplinary meeting, a responsible person is appointed to create the labels for the premises/activities” (HCSO-3)	8
	“Homepage will be adapted by external administrator after team discussion” (GPP-2)	“[Name] is in contact with the housekeeper. We will receive an update after two weeks on how to best improve measures to provide easy access” (HCSO-4)	
	“We create a pictogram and hang it on the wall in front of the toilet to make it clearly visible from all angles where the toilet is located” (GPP-3)	“Improve the forwarding to the department, which is responsible for health literacy issues (within the center)” (HCSO-6)	
	“We will contact tele-search, since entry of the tele-search platform is not correct” (GPP-4)	“Hand out flyer to medical doctors for people with dementia” (HCSO-6)	
2. Communicating in plain and easy-to-understand language	“Internal training at team meetings by medical doctors” (GPP-1)	“Implementation and usage of the measurements are being evaluated at the summer meeting in 2021” (HCSO-3)	5
	“Internal forms are distributed to MPAs and processed. After approval by the medical doctors, the forms will be used more frequently again” (GPP-2)		
	“Create guideline and file it in the practice manual for everyone to refer to” (GPP-3)		
	“Restock brochures and draft fact sheets” (GPP-4)		
3. Promoting health literacy of users	“Communicate existing information more actively to patients” (GPP-1)	“Recruiting another PH staff member so that all HW clients have a reference person” (HCSO-2)	5
	“Plan information event/open day in cooperation with partner organizations, e.g., HCSO” (GPP-1)	“Create an information folder on health literacy and store it in the team office, accessible to every staff member” (HCSO-6)	
	“In December, we will plan a patient consultation on the subject of diabetes” (GPP-2)		
4. Promoting health literacy of staff members	“Guidelines by medical doctors including evidence-based sources of information” (GPP-2)	“The topic of health literacy was forwarded to the management level. More information will follow” (HCSO-2)	9
	“Defining creditable sources at the meeting with the CEO, so everyone shares the same information with patients” (GPP-3)	“Each staff member can provide an input on OHL. Every two months there will be a technical input. Start in January 2021” (HCSO-3)	
		“Providing guidelines on our internal digital platform, implementation in team meetings” (HCSO-4)	
	“Adapting staff communication guidelines” (GPP-4)	“Include a health literacy refresher in our team meeting, where a staff member introduces a specific topic. In each team meeting, it is re-determined what topic will be discussed next time and who will present it” (HCSO-6)	
		“Work supervision has already taken place, improving feedback culture” (HCSO-6)	
		“Case discussions are considered to take place twice a year” (HCSO-6)	
5. Incorporating health literacy into the management and organizational structure	“Demonstrating practices 1:1, viewed by MPA and management” (GPP-4)	“We regularly discuss the topic of staying healthy and strengthening one’s own health in sessions. To do this, we use the battery method from 0 to 10 and regularly reflect on our own health status to support each other” (HCSO-1)	2
6. Promoting health literacy at care interfaces, networks and further activities of the organization	“Providing a link list on the homepage and an overview list with relevant links for staff members” (GPP-4)	“We organize a training for our team to improve health literate communication in the organization” (HCSO-1)	4
		“Someone from the center or organizational development department will be appointed to take part in the reports and pass on feedback/information to the other teams” (HCSO-3)	
		“Increase participation at organizational offers regarding health literacy from 2021 onwards” (HCSO-3)	
**All dimensions**			**33**

## Data Availability

The data presented in this study are available on request from the corresponding author. The data are not publicly available to ensure the privacy of our practice partners.
